# MAFLD increases CKM syndrome severity: a NHANES-based cross-sectional study

**DOI:** 10.1007/s10238-025-01997-1

**Published:** 2025-12-26

**Authors:** Alaa M. Mostafa, Yasser Fouad, Ziayn Pan, Shereen Abdel Alem, Mohamed AbdAllah, Eman Abdelsameea, Mohammed Eslam

**Affiliations:** 1 Department of Gastroenterology, Hepatology and Endemic Medicine, Faculty of Medicine, Minia University, Main Road, Minia, 11432 Egypt; 2 Storr Liver Centre, Westmead Institute for Medical Research, Westmead Hospital and University of Sydney, Sydney, NSW Australia; 3 Endemic Medicine and Hepatology Department, Faculty of Medicine, Cairo University, Cairo, Egypt; 4 Medical Research Division, National Research Center, Giza, Egypt; 5 Department of Hepatology and Gastroenterology, National Liver Institute, Menoufia University, Menoufia, Egypt

**Keywords:** MAFLD, CKM syndrome, Fibrosis, NHANES

## Abstract

The cardiovascular-kidney-metabolic (CKM) syndrome is a newly proposed clinical concept that emphasizes the interconnected burden of renal and cardiometabolic dysfunction. However, the impact of metabolic dysfunction-associated fatty liver disease (MAFLD) within the CKM staging framework is still unknown. This cross-sectional study utilized data from the U.S. National Health and Nutrition Examination Survey (NHANES) from 2017 to 2020. Participants aged 18–80 were categorized by CKM staging was applied based on the 2023 American Heart Association classification, MAFLD status, and liver fibrosis was assessed by transient elastography and non-invasive scores. Logistic regression was used to identify independent predictors of advanced CKM stages. Of 6167 participants, 5695 (92.3%) met the CKM Framework, with 3137 (55.1%) having MAFLD (CKM-MAFLD). Those with CKM-MAFLD exhibited significantly higher cardiometabolic risks, particularly for cardiovascular events. Late CKM stages showed a higher prevalence of MAFLD (57.7% vs 53.2%, p < 0.001) and greater steatosis and fibrosis. MAFLD was an independent predictor of CKM risk (OR: 1.2, 95% CI: 1.03–1.5, P 0.04), an effect was more profound in those with liver fibrosis (OR: 1.36, p-value 0.004). MAFLD could be a crucial predictor in CKM risk stratification, emphasizing the need for hepatic evaluation in these frameworks. Early identification of MAFLD-related dysfunction can lead to targeted interventions across the liver-heart-kidney continuum.

**Supplementary Information:**

The online version contains supplementary material available at 10.1007/s10238-025-01997-1.

## Introduction

In recognition of the complex interrelationship between cardiovascular, renal, and metabolic dysfunctions, the American Heart Association (AHA) recently introduced the framework of cardiovascular-kidney-metabolic (CKM) syndrome as a new concept for the ongoing relationship between metabolic stress and multiorgan damage, while proposing a novel staging system for earlier risk identification and integrated care [1].

Metabolic dysfunction-associated fatty liver disease (MAFLD) is a primary liver disease that affects nearly a quarter of the global population and is increasingly recognized as a systemic disease, with a multitude of extra-hepatic manifestations, including association with cardiovascular and renal-related morbidity and mortality [2–6]. Notably, cardiovascular disease remains the primary cause of death among individuals with MAFLD, emphasizing the implications for risk assessment and management strategies, beyond the hepatic assessment.

Despite this, the role of MAFLD in this multiorgan metabolic dysfunction remains largely unexplored, and the role of MAFLD within the CKM framework is unknown. This study aims to assess the impact of integrating MAFLD on the risk and severity of the recent CKM framework.

## Patients and methods

### Study design

This cross-sectional study used the NHANES database (2017–2020) cycle. This survey is part of the Centers for Disease Control and Prevention’s archiving program, which aims to assess the nutritional and overall health of people in the United States [7].

### Study population

**Fig. 1:**
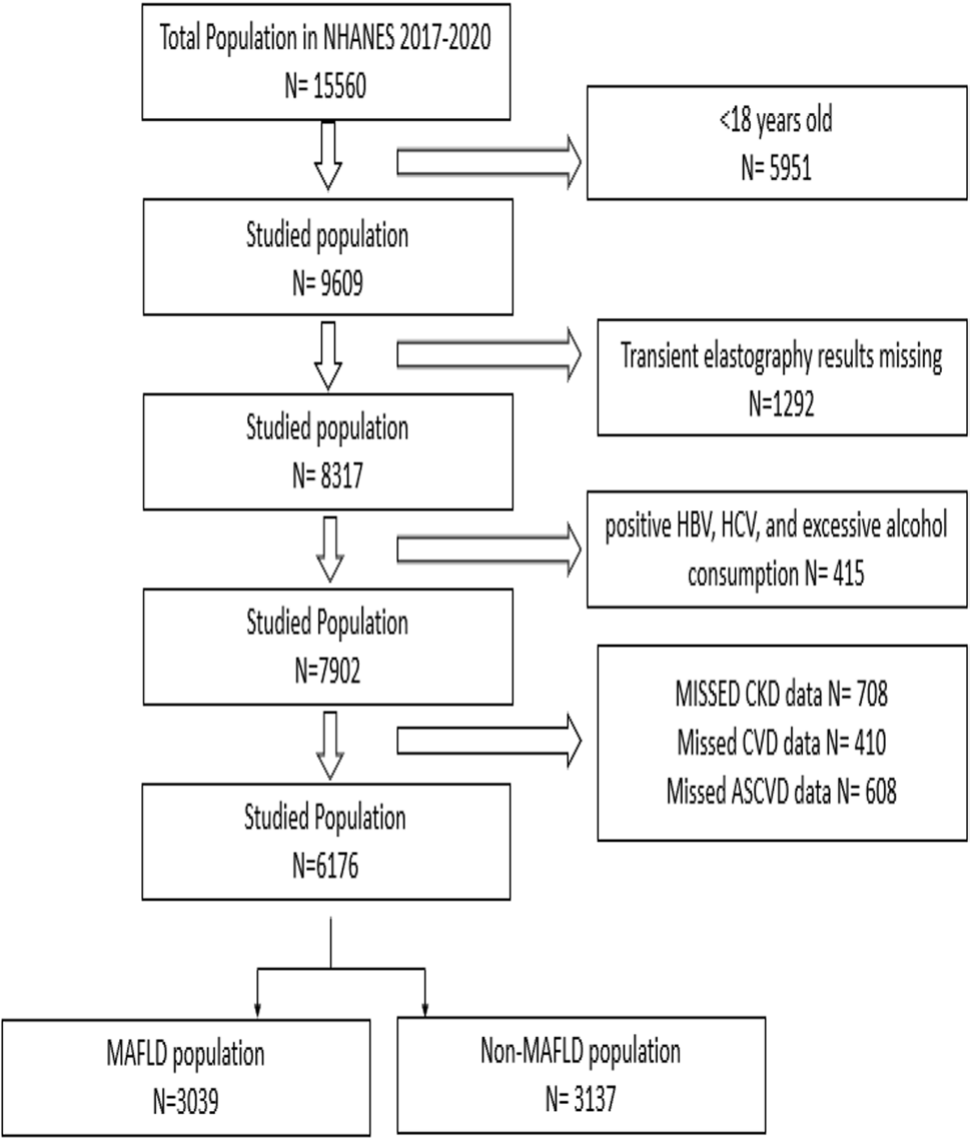
Flowchart of the NHANES studied population

Participants aged 18 to 80 years were included in the study. Exclusion criteria include individuals with missing or incomplete data on transient elastography, CKD, and CVD. Individuals who were positive for hepatitis C antibody and confirmed by detectable HCV RNA level or who were positive for hepatitis B surface antigen (HBs-Ag) or had significant alcohol consumption [> 2 drinks per day for men and > 1 per day for women every or nearly every day] were excluded from the study (Fig. 1).

### Definition of MAFLD and fibrosis assessment

MAFLD is identified by the presence of hepatic steatosis, which is assessed using Vibration Controlled Transient Elastography (VCTE), alongside metabolic dysregulation. This dysregulation can include overweight or obesity, type 2 diabetes mellitus, or lean with two or more components of metabolic syndrome.

VCTE measurements were acquired at the NHANES Mobile Examination Center (MEC) using the FibroScan® model 502 V2 Touch system. Probe selection (M or XL) was determined by the NHANES protocol based on chest wall thickness.

To ensure data quality and reliability, all VCTE procedures adhered to strict NHANES protocols. These included comprehensive technician training, regular equipment calibration, and real-time expert review of scans. A valid elastography examination was defined by a minimum of 10 valid measurements, with an interquartile range to median ratio (IQR/M) of less than 30%. Scans that did not meet these quality control criteria were considered unreliable and were excluded from the final analysis.

A median Controlled Attenuation Parameter (CAP) cut-off of ≥ 264 dB/m was used to define steatosis grade S1, and a liver stiffness measurement (LSM) cut-off of ≥ 8 kPa indicated significant fibrosis (F2). Significant fibrosis was classified as LSM ≥ 8 kPa [2, 8].

Detailed information on the VCTE protocol and quality assurance procedures can be found on the NHANES website (https://www.cdc.gov/nchs).

Additionally, the Fibrosis-4 (FIB-4) index and the NAFLD Fibrosis Score (NFS) were calculated as non-invasive measures for assessing the probability of advanced fibrosis. For FIB-4, a cut-off of ≥ 1.3 was used to indicate significant fibrosis (moderate-to-high probability). For NFS, advanced fibrosis was classified into three categories based on validated cut-offs: < -1.455 for a low probability, -1.455 to 0.676 for an indeterminate probability, and > 0.676 for a high probability. Notably, the low cut-off of NFS (< -1.455) was particularly effective for accurately excluding advanced fibrosis. [9, 10].

### CKM staging framework

CKM syndrome staging was applied to all participants with MAFLD. The CKM stages are categorized according to AHA (correspondence in NHANES dataset) as follows:

Stage 0: Individuals with no metabolic abnormalities,

Stage 1: Individuals who are either overweight/obese (BMI) or have insulin resistance (represented by HOMA-IR).

Stage 2: Individuals who exhibit additional metabolic risk factors, such as hypertension (HTN), dyslipidemia, diabetes mellitus (DM), or early renal impairment (represented GFR and A/C ratio).

Stage 3: Individuals with subclinical cardiovascular disease (assessed by ASCVD score) or advanced chronic kidney disease (CKD).

Stage 4: Individuals with established clinical CVD, including conditions like coronary artery disease, stroke, or heart failure.

For comparison, participants were classified into two groups: early stage (CKM stages 1–2) and late stage (CKM stages 3–4) [1].

### Cardiovascular and renal risk assessments

CVD evidence was reported from medical condition records collected through a home questionnaire administered by trained interviewers using a Computer-Assisted Personal Interview (CAPI) system. The questionnaire included questions such as, “*Have you ever been told you had: angina* (MCQ160d)*, coronary heart disease (*MCQ160c)*, a heart attack (*MCQ160e)*, or a stroke (*MCQ160f)?” Subclinical cardiovascular risk was evaluated using established Atherosclerotic Cardiovascular (ASCVD) risk calculators appropriate for individuals aged 20 to 79 years.

On the other hand, chronic kidney disease (CKD) risk was assessed using the estimated glomerular filtration rate (eGFR) and the albumin-to-creatinine ratio (ACR). CKD risk was stratified according to KDIGO guidelines into categories of low, moderate, high, and very high risk. [11].

### Demographic and biochemical variables collected

Demographic data, including age, sex, and ethnicity, as well as anthropometric indices such as body mass index (BMI) and waist circumference, were reported. Overweight or obesity was defined based on BMI values of ≥ 25 kg/m^2^ (or ≥ 23 kg/m^2^ for Asians) and waist circumference measurements of ≥ 102 cm for Caucasian men, ≥ 88 cm for Caucasian women, and ≥ 90 cm for Asian men and women, or ≥ 80 cm for Asian women. Hypertension was identified as the blood pressure was ≥ 130/85 mmHg or if the individual has been told by a doctor that they have hypertension, as reported in the questionnaire (BPQ030). Prediabetes and diabetes were defined according to the questionnaire, where respondents indicated if a doctor had informed them, they had diabetes (DIQ010).

Laboratory data included fasting blood sugar (FBS), hemoglobin (Hb)A1C, the Homeostasis Model Assessment of Insulin Resistance (HOMA-IR) score, plasma high-sensitivity C-reactive protein (hs-CRP) levels, cholesterol, triglycerides, low-density lipoprotein (LDL), high-density lipoprotein (HDL), total leukocyte count (TLC), platelet count, alanine aminotransferase (ALT), aspartate aminotransferase (AST), serum albumin, uric acid, alkaline phosphatase, serum creatinine, estimated glomerular filtration rate (eGFR), and albumin-to-creatinine ratio (ACR).

### Statistical analysis

Continuous variables are presented as mean ± standard deviation, while categorical variables are shown as frequency (%). Group comparisons were performed using independent t-tests or chi-square tests, as appropriate. Logistic regression analysis was carried out to identify predictors of severe CKM stages, adjusting for pre-specified confounders including age, sex, smoking status, ethnicity, and cardiovascular risk (as defined by ASCVD score where applicable). Odds ratios (OR) with 95% confidence intervals (CI) were reported. A p-value of less than 0.05 was considered statistically significant. Multicollinearity among predictors in the logistic regression models was assessed using Variance Inflation Factors (VIF).

## Results

### Baseline characteristics of the NHANES population

The baseline characteristics of the cohort study, categorized into patients who fulfilled MAFLD criteria (N = 3,137, 50.8%) and those who did not (N = 3,039, 49.2%), are shown in Table 1.

**Table 1:** Baseline characteristics of the NHANES population (2017–2020): MAFLD and non-MAFLD

Variable	All N 6176	Non-MAFLD N 3039	MAFLD N 3137	p-value
Age (18–80)	50.7 ± 17.3	48.6 ± 18.3	52.7 ± 15.9	< 0.001
Male	2998 (48.5)	1333 (43.8)	1665 (53.1)	< 0.001
Smoking	1009 (16.3)	517 (17)	492(15.7)	< 0.001
Non-Hispanic White	2128 (34.4)	1002(32.9)	1126(35.9)	0.01
BMI (Kg/mm^2^)	30.4 ± 7.6	26.8 ± 5.9	33.8 ± 7.4	< 0.001
Waist Circumference (cm)	101.7 ± 17.3	92.6 ± 14.3	110.7 ± 15.2	< 0.001
Hypertension	3491 (56.5)	1448(47.6)	2043(65.1)	< 0.001
Diabetic Mellitus	984 (15.9)	271(8.9)	713(22.7)	< 0.001
Prediabetes	205 (3.3)	75(2.5)	130(4.1)	< 0.001
FBS mmol/L	6.3 ± 2.1	5.8 ± 1.6	7 ± 2.6	< 0.001
HbA1C mmol	5.9 ± 1.2	5.6 ± 0.8	6.2 ± 1.3	< 0.001
HOMA-IR	4.5 ± 8.2	2.8 ± 5.6	6.8 ± 10.4	< 0.001
HS-CRP mg/L	4.2 ± 8.6	3.2 ± 7.8	5.1 ± 9.1	< 0.001
Dyslipidemia	2180 (35.3)	731(24.1)	1449 (46.2)	< 0.001
Triglyceride mg/dL	111.3 ± 98.2	89.7 ± 64.7	141.4 ± 125.4	< 0.001
Cholesterol mg/dL	185.7 ± 40.4	183.4 ± 39.7	187.9 ± 40.9	< 0.001
LDL mg/dL	111.2 ± 35.8	109.8 ± 35.4	113 ± 36.3	0.03
HDL mg/dL	52.5 ± 15.3	57.1 ± 15.6	48.1 ± 13.6	< 0.001
TLC µL	7.2 ± 2.3	6.7 ± 2.4	7.6 ± 2.1	< 0.001
Platelet (10^9^)	246.8 ± 65.01	242.8 ± 63.2	250.8 ± 66.5	< 0.001
ALT IU/L	22.1 ± 15.8	18.3 ± 11.4	25.8 ± 18.5	< 0.001
AST IU/L	21.3 ± 11.4	20.1 ± 8.9	22.4 ± 13.2	< 0.001
S albumin g/dL	4.1 ± 0.3	4.1 ± 0.3	4.03 ± 0.3	< 0.001
S. Uric acid mg/dL	5.4 ± 1.45	5.1 ± 1.4	5.8 ± 1.5	< 0.001
Alkaline Phosphatase IU/L	78.4 ± 26.2	75 ± 26.7	81.6 ± 25.3	< 0.001
S. Creatinine mg/dL	0.9 ± 0.4	0.8 ± 0.3	0.8 ± 0.4	0.03
ACR mg/g	5.7 ± 41.6	5.4 ± 46.7	6.1 ± 35.9	< 0.001
eGFR ml/min/1.73m^2^	91.3 ± 22.4	92.1 ± 22.6	90.4 ± 22.2	< 0.001
BUN mg/dL	14.9 ± 5.7	14.6 ± 5.4	15.2 ± 6.01	< 0.001
Complement 3 mg/dL	25.5 ± 2.4	25.6 ± 2.4	25.3 ± 2.4	< 0.001
CAP dB/m	271.4 ± 62.7	220.6 ± 38.1	320.8 ± 37.4	< 0.001
LSM kPa	6.05 ± 5.2	5.1 ± 4.3	6.9 ± 5.9	< 0.001
Fib-4	1.07 ± 0.8	1.08 ± 0.7	1.07 ± 0.9	0.7
NFS	-1.5 ± 1.6	-1.8 ± 1.4	-1.2 ± 1.5	< 0.001
ASCVD (20–79)	2.4 ± 41.3	0.7 ± 37.4	11.8 ± 43.9	< 0.001

WC: waist circumference; FBS: fasting blood sugar; HbA1C: glycated hemoglobin; HOMA-IR: homeostatic model assessment for insulin resistance; HS-CRP: high sensitivity C-reactive protein test; LDL: low density lipoprotein, HDL: high-density lipoprotein; TLC: total leucocyte count; ALT: alanine transferase; AST: aspartate transferase; CAP: controlled attenuation parameter; LSM: liver stiffness measurement; FIB-4: fibrosis-4 index; NFS: NAFLD fibrosis score; ASCVD: Atherosclerosis cardiovascular disease score.

Individuals with MAFLD are significantly older (52.7 ± 15.9 years, p < 0.001) and have a higher proportion of non-Hispanic Whites (35.9% vs. 32.9%, p < 0.01). These individuals exhibit metabolic dysfunction, higher BMI (33.8 ± 7.4 kg/m^2^), waist circumference (110.7 ± 15.2 cm), and increased prevalence of diabetes (22.7% vs. 8.9%), hypertension (65.1% vs. 47.6%), and dyslipidemia (46.2% vs. 24.1%), all with p < 0.001.

Additionally, inflammatory and liver-related markers were significantly elevated in the MAFLD group, including liver enzymes, lipid profile, Hs-CRP, uric acid, alkaline phosphatase, and lower platelet count, p < 0.001 for all comparisons. Moreover, MAFLD patients had a notably higher atherosclerotic cardiovascular disease (ASCVD) risk score (11.8 ± 43.9 vs. 0.7 ± 37.4, p < 0.001).

### MAFLD is associated with increased CKM risk

After applying the CKM score, 5695 patients met the CKM criteria. Further subgrouping of the CKM patients into those who exhibited MAFLD changes (CKM-MAFLD 3137 (55.1)) and others without MAFLD (CKM-non-MAFLD, 2558 (44.9), Table 2.

**Table 2:** Comparative results among CKM framework: CKM-MAFLD and CKM non-MAFLD

Variable	CKM 5695	CKM NON-MAFLD 2558 (44.9)	CKM MAFLD 3137 (55.1)	p-value
Age (18–80)	51.8 ± 17	50.8 ± 18	52.7 ± 15.9	< 0.001
Male	2808	1143 (44.7)	1665 (53.1)	< 0.001
Smoking	911	419 (16.4)	492 (15.7)	0.02
Non-Hispanic White	1941	815 (31.9)	1126 (35.8)	0.001
BMI (Kg/mm^2^)	31.1 ± 7.4	27.9 ± 5.9	33.8 ± 7.4	< 0.001
Waist circumference (cm)	103.8 ± 16.4	95.4 ± 13.6	110.7 ± 15.2	< 0.001
Hypertension	3480	1437 (56.2)	2043 (65.1)	< 0.001
Diabetic Mellitus	1298 (22.8)	334 (13.1)	964 (30.7)	< 0.001
FBS (mmol/L)	6.4 ± 2.1	5.9 ± 1.6	7 ± 2.6	< 0.001
HBAIC mmol	5.9 ± 1.2	5.6 ± 0.9	6.2 ± 1.3	< 0.001
HOMA-IR	4.7 ± 8.5	3.03 ± 5.9	6.8 ± 10.4	< 0.001
HS-CRP (mg/L)	4.4 ± 8.8	3.5 ± 8.4	5.1 ± 9.1	< 0.001
Triglyceride (mg/dL)	115.3 ± 100.5	94.1 ± 67.2	141.4 ± 125.4	< 0.001
Cholesterol (mg/dL)	186.5 ± 40.8	184.6 ± 40.5	187.9 ± 40.9	< 0.001
LDL (mg/dL)	112.1 ± 36	111.3 ± 35.8	113 ± 36.3	0.09
HDL (mg/dL)	51.5 ± 14.9	55.7 ± 15.4	48.1 ± 13.6	< 0.001
ALT (IU/L)	22.6 ± 16.1	18.6 ± 11.5	25.8 ± 18.5	< 0.001
AST (IU/L)	21.4 ± 11.5	20.1 ± 8.9	22.4 ± 13.2	< 0.001
Alkaline phosphatase	79.3 ± 25.2	76.6 ± 25	81.6 ± 25.3	< 0.001
S Uric Acid (mg/dL)	5.5 ± 1.4	5.2 ± 1.4	5.8 ± 1.5	< 0.001
S. Creatinine (mg/dL)	0.9 ± 0.4	0.9 ± 0.4	0.9 ± 0.4	0.4
BUN (mg/dL)	15.1 ± 5.9	14.9 ± 5.6	15.2 ± 6	0.04
CAP (dB/m)	277.1 ± 61	223.6 ± 37.2	320.7 ± 37.4	< 0.001
LSM (kPa)	6.2 ± 5.4	5.3 ± 4.6	6.9 ± 5.9	< 0.001
F ≥ 2	712 (12.5)	132 (5.2)	580 (18.5)	< 0.001
Fib-4	1.1 ± 0.9	1.1 ± 0.7	1.1 ± 0.9	0.01
NFS	-1.4 ± 1.5	-1.6 ± 1.5	-1.2 ± 1.5	< 0.001
ASCVD	4.1 ± 42.5	1.3 ± 39.6	11.8 ± 43.9	< 0.001

WC: waist circumference; FBS: fasting blood sugar; HbA1C: glycated hemoglobin; HOMA-IR: homeostatic model assessment for insulin resistance; HS-CRP: high sensitivity C-reactive protein test; LDL: low density lipoprotein, HDL: high-density lipoprotein; TLC: total leucocyte count; ALT: alanine transferase; AST: aspartate transferase; CAP: controlled attenuation parameter; LSM: liver stiffness measurement; FIB-4: fibrosis-4 index; NFS: NAFLD fibrosis score; ASCVD: Atherosclerosis cardiovascular disease score.

CKM-MAFLD patients had a notably worse cardiometabolic risk profile than those with CKM alone. They tended to be older (52.7 vs 50.8, p < 0.001), more often male (53.1 vs 44.7, p < 0.001), and had higher BMI (33.8 vs 27.9, p < 0.001) and waist circumference (110.7 vs 95.4, p < 0.001). Hypertension and type 2 diabetes were more common in the CKM-MAFLD group, who also showed higher fasting glucose, HbA1c (6.2 vs 5.6 mmol, p < 0.001), HOMA-IR (6.8 vs 3.03, p < 0.001), triglycerides (141.4 vs 94.1, p < 0.001), HS-CRP (5.1 vs 3.5 mg/L), and s uric acid (5.8 vs 5.2, p < 0.001).

### Cardiorenal risk in CKM patients with and without MAFLD

**Fig. 2:**
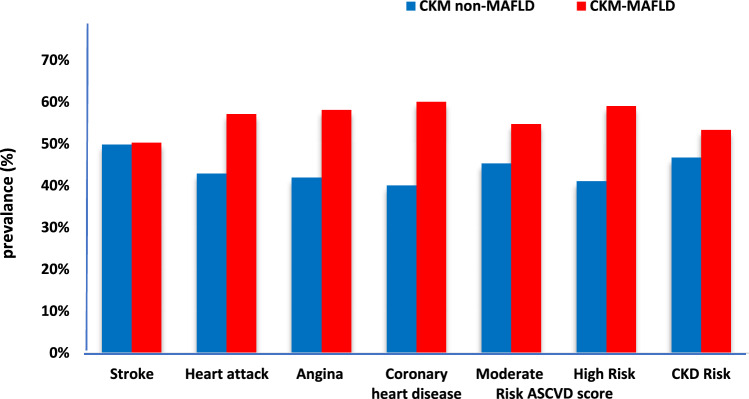
Cardiorenal risk in CKM patients with and without MAFLD

Consequently, those patients who fulfilled CKM-MAFLD criteria had a higher burden of cardiovascular risk and events than those with CKM-non-MAFLD, as shown in Fig. 2. Across the ASCVD risk categories, those patients demonstrated a greater proportion of moderate (54.7% vs 45.3%) to high-risk (58.6% vs 41.4%) scores. Consistently, the prevalence of angina (58.1% vs 41.9%), coronary heart disease (60% vs 40%), and prior heart attack (57.1% vs 42.9%) was higher among CKM-MAFLD. In addition to the higher CKD in that population (53.3 vs 46.7).

Additionally, in a multivariable logistic regression analysis, after adjusting for confounders, MAFLD was identified as an independent risk factor for CKM risk (OR: 1.2, 95% CI: 1.03–1.5, p = 0.04), Table 3. Notably, all variables included in the model exhibit VIF below 5, indicating no significant multicollinearity.

**Table 3:** Multivariate logistic regression for CKM risk predictors

	OR (95% CI)	P-value
Age	1.01 (1.01–1.02)	< 0.001
Sex	1.09 (1.01–1.27)	0.3
Smoker	1.08 (0.9–1.3)	0.3
Non-Hispanic White	1.17 (0.9–1.4)	0.06
BMI	0.96 (0.95–0.97)	< 0.001
Diabetes	1.4 (1.1–1.7)	0.04
Hypertension	1.04 (0.8–1.3)	0.6
MAFLD	1.2 (1.03–1.5)	0.04
LSM	1 (0.98–1.02)	0.7

BMI: body mass index; MAFLD: metabolic-associated fatty liver disease; LSM: liver stiffness measurements.

### MAFLD is associated with increased CKM severity

Further stratification of CKM patients into early (Stage 1,2 n = 3273) and late (Stage 3,4 n = 2422) CKM, Table 4. Identifying late CKM with a higher risk of CKD and CVD. Notably, MAFLD prevalence was higher in late CKM stages (57.7% vs 53.2%) than in early stages, with corresponding increases in non-invasive markers, CAP (279.4 vs 275.4), LSM (6.2 vs 5.9), Fib-4 (1.2 vs 1), and NFS (-1.2 vs -1.5).

**Table 4:** Comparison between early and late CKM Stage

	Early CKM N 3273	Late CKM N 2422	p-value
Age (18–80)	49.6 ± 16.7	54.8 ± 16.8	< 0.001
Male	1532 (46.8)	1276 (52.7)	< 0.001
Smoking	504 (15.4)	407 (16.8)	0.3
Non-Hispanic White	1030 (31.5)	911 (37.6)	< 0.001
BMI (Kg/mm^2^)	31.7 ± 7.9	30.3 ± 6.3	< 0.001
Waist Circumference	104.4 ± 17.6	102.8 ± 14.5	< 0.001
Hypertension	1910 (58.4)	1570 (64.8)	< 0.001
Diabetic Mellitus	457 (13.9)	523 (21.6)	< 0.001
Prediabetes	121 (3.7)	84 (1.9)	0.6
FBS (mmol/L)	6.3 ± 1.9	6.5 ± 2.3	< 0.001
HBAIC (mmol)	5.9 ± 1.09	6.1 ± 1.3	< 0.001
HOMA-IR	4.5 ± 8.8	4.5 ± 7.4	0.3
ACR (mg/g)	3.4 ± 14.8	9.7 ± 63.8	< 0.001
eGFR (ml/min/1.73m^2^)	94.1 ± 21.4	86.9 ± 23.3	< 0.001
BUN (mg/dL)	14.5 ± 4.9	15.7 ± 6.7	< 0.001
HS-CRP (mg/L)	4.4 ± 9.3	4.3 ± 7.9	0.009
Triglyceride (mg/dL)	116.3 ± 90.6	114.3 ± 110	0.002
Cholesterol (mg/dL)	188.1 ± 39.8	184.2 ± 41.8	0.008
LDL (mg/dL)	113.9 ± 35.4	110.1 ± 36.5	0.08
HDL (mg/dL)	51.5 ± 14.9	51.4 ± 14.8	< 0.001
Platelet (10^9^)	251.7 ± 65.6	241.7 ± 65.4	< 0.001
ALT (IU/L)	23.3 ± 17.4	21.6 ± 14.2	0.6
AST (IU/L)	21.6 ± 12.4	21 ± 10.1	0.8
S albumin (g/dL)	4.05 ± 0.3	4.04 ± 0.3	< 0.001
Alkaline phosphatase	78.4 ± 24.3	80.6 ± 26.4	< 0.001
S Uric Acid (mg/dL)	5.4 ± 1.5	5.5 ± 1.4	< 0.001
S. Creatinine (mg/dL)	0.8 ± 0.23	0.9 ± 0.5	0.5
Complement 3 (mg/dL)	25.4 ± 2.4	25.5 ± 2.4	0.05
MAFLD	1740 (53.2)	1397 (57.7)	< 0.001
CAP (dB/m)	275.4 ± 63.3	279.4 ± 57.8	< 0.001
LSM (kPa)	5.9 ± 4.8	6.2 ± 5.7	< 0.001
Fib-4	1 ± 0.6	1.2 ± 1.07	< 0.001
NFS	-1.5 ± 1.5	-1.2 ± 1.6	< 0.001

WC: waist circumference; FBS: fasting blood sugar; HbA1C: glycated hemoglobin; HOMA-IR: homeostatic model assessment for insulin resistance; HS-CRP: high sensitivity C-reactive protein test; LDL: low density lipoprotein, HDL: high-density lipoprotein; TLC: total leucocyte count; ALT: alanine transferase; AST: aspartate transferase; CAP: controlled attenuation parameter; LSM: liver stiffness measurement; FIB-4: fibrosis-4 index; NFS: NAFLD fibrosis score; ASCVD: Atherosclerosis cardiovascular disease score.

### Risk factors of cardiovascular risk in CKM

Multivariable logistic regression showed that older age (OR: 1.02; 95% CI: 1.02–1.03, p < 0.001) was linked to higher cardiovascular risk in CKM-MAFLD patients. Diabetes (OR: 1.26; 95% CI: 1.05–1.51, p 0.01), higher serum creatinine (OR: 1.48; 95% CI: 1.1–1.9, p 0.005), and Lower platelet counts (OR: 0.99; 95% CI: 0.99–1, p 0.04) were also significant predictors. Higher fibrosis (NFS OR: 1.36; 95% CI: 1.1–1.68, p 0.004) was associated with increased cardiovascular risk among the CKM criteria, Table 5.

**Table 5:** Predictors of Cardiovascular risk in CKM patients

	OR (95%CI)	P-value
Age	1.02 (1.02–1.03)	< 0.001
Sex	1.1 (0.91–1.29)	0.3
Non-Hispanic White	1.16 (0.89–1.35)	0.07
BMI	1.4 (0.78–2.53)	0.2
Diabetic	1.26 (1.05–1.51)	0.01
Hypertension	1.09 (0.9–1.3)	0.3
Platelet	0.99 (0.99–1)	0.04
Alkaline phosphatase	1.002 (0.99–1.01)	0.3
NFS	1.36 (1.1–1.68)	0.004
Serum creatinine	1.48 (1.1–1.9)	0.005

MAFLD: metabolic-associated fatty liver disease; BMI: body mass index; NFS: NAFLD fibrosis score.

## Discussion

In this large data from NHANES 2017–2020, utilizing the newly proposed AHA CKM classification, we found a robust link between MAFLD, CKM staging, and related severity. Integrating MAFLD status into the CKM staging categories, we found a progressive increase in cardiorenal risk, characterized by higher ASCVD scores and a higher prevalence of CKD and evidence of CVD as the CKM stage advances in those with MAFLD compared with CKM without. MAFLD was consistently identified as an independent associate of CKM risk strata. Moreover, hepatic fibrosis was considered a risk factor of cardiovascular risk as well as renal and metabolic dysfunction among the CKM new concept.

The CKM syndrome framework, recently introduced by the AHA, represents a significant advancement in understanding the interconnection between metabolic risk factors, renal impairment, and cardiovascular disease within a unified continuum of pathophysiology [1]. This framework features a staging system aimed at promoting integrated prevention, early intervention, and risk stratification across multiple organ systems. However, despite the extensive evidence linking MAFLD to both cardiovascular and kidney outcomes, hepatic involvement is not explicitly included in the current CKM classification.

Previous population-based studies have consistently demonstrated that MAFLD is alarmingly prevalent in Western countries, affecting between 38 and 55% of adults [12, 13]. A condition, recognized now as a complex multisystem disorder, far exceeding the confines of a typical liver disease [14, 15]. Notably, research highlights that hepatic fibrosis serves as the critical histological predictor of long-term health outcomes. It significantly forecasts cardiovascular events, chronic kidney disease, and mortality, regardless of traditional cardiometabolic risk factors [16–18].

In our cohort, MAFLD was found to be independently associated with CKM risk and its severity-related cardiorenal risk, which is consistent with recent evidence suggesting that MAFLD poses a greater risk for CVD and kidney disease than non-MAFLD phenotypes [5, 15]. The increased risk observed in our cross-sectional cohort aligns with existing mechanistic hypotheses from external research. For instance, hepatic insulin resistance is understood to initiate hyperinsulinemia, increased gluconeogenesis, and systemic glucose dysregulation [18]. These metabolic disturbances are thought to accelerate atherosclerosis and contribute to renal microvascular injury [19, 20]. Concurrently, hepatocyte-derived pro-inflammatory cytokines (e.g., TNF-α, IL-6) and pro-atherogenic lipoproteins are known to propagate endothelial dysfunction, thereby fostering a ‘hepato-cardio–renal axis’ of injury [21]. Moreover, emerging evidence highlights that the hepatic immune microenvironment may influence systemic inflammation and dysfunction across various organs. Changes in antigen presentation through MHC class I and II pathways could thus impact both local liver damage and systemic immune signaling, potentially exacerbating metabolic and cardiovascular risks [22]. Despite this accumulating evidence, current CKM staging omits hepatic metrics, which may lead to an underestimation of risk in this high-burden subgroup.

Furthermore, Participants with MAFLD were overrepresented in the later stages of CKD and exhibited a higher prevalence of clinical cardiovascular events, particularly in the presence of hepatic fibrosis. This supports previous longitudinal data indicating that hepatic fibrosis is the key histological predictor of adverse outcomes and aligns with previous studies showing that the stage of fibrosis independently predicts both liver-related and non-liver-related mortality in patients with MAFLD [16, 17]. Conversely, some population studies have highlighted the importance of metabolic comorbidities over histological severity in predicting outcomes [18]. Supporting the hypothesis of the additive risk effect of both fibrosis and metabolic dysfunction.

Interestingly, we found liver fibrosis was independently linked to cardiovascular risk in advanced stages of CKM, in line with evidence that these scores can predict cardiovascular and renal events, likely reflecting the systemic nature of advanced fibrotic disease [23, 24]. Our findings suggest that fibrosis may represent a cumulative metabolic and inflammatory driver affecting cardiovascular and renal dysfunction. Subsequently, MAFLD, particularly in the presence of hepatic fibrosis, appears to be associated with a crucial role in determining multisystem risk within the cardiovascular-kidney-metabolic continuum [24].

Furthermore, we observed a stepwise increase in cardiovascular and renal risk among individuals with CKM-MAFLD than those with CKM-non-MAFLD, supporting the idea that the liver is an active participant in systemic metabolic disease rather than a passive bystander [14, 15]. This perspective aligns with recent proposals to expand the current CKM framework into a cardiovascular-renal-hepatic-metabolic (CRHM) model, formally acknowledging the liver’s role in multisystem dysfunction [25]. Mechanistically, hepatic insulin resistance, low-grade inflammation, and the release of pro-atherogenic lipids from the damaged liver create a plausible link to vascular injury and renal impairment. These mechanisms may underline the observed associations; longitudinal studies are needed to confirm causality. Therefore, by addressing this significant gap, we can enhance risk stratification, facilitate the early identification of high-risk phenotypes, and drive the integration of liver health metrics into comprehensive CKM assessment models. Although the observed odds ratios for MAFLD predicting CKM severity were modest (e.g., OR: 1.2), their statistical significance across a large, representative cohort underscores a consistent association. In the context of a highly prevalent condition like MAFLD and the broad public health implications of CKM syndrome, even a small but consistent increase in risk can translate to a substantial population burden and warrants clinical attention.

The major strength of this study is the utilization of a large, nationally representative cohort from NHANES 2017–2020, which allows for the generalization of our findings to the U.S. adult population. Liver disease status was objectively assessed using VCTE, providing precise and non-invasive quantification of both steatosis and fibrosis severity, enabling us to robustly stratify MAFLD according to fibrosis stage, which is crucial given its prognostic significance for cardiovascular and renal outcomes. Likewise, applying the recently proposed AHAs CKM staging framework to a MAFLD population, with and without fibrosis, is a novel aspect of this study that offers an integrated perspective on multi-organ risk.

Several limitations should be acknowledged in this study. First, the cross-sectional design of NHANES prevents us from causal inferences. Besides, the exclusion of patients with valid records of VCTE may underestimate the effect of MAFLD on the CKM framework by underestimation of individuals with more advanced liver disease. While VCTE is a validated non-invasive method for assessing steatosis and fibrosis, its accuracy may be diminished in individuals with high BMI or significant hepatic steatosis, which could result in misclassification of fibrosis. Additionally, the NHANES transient elastography protocol excludes individuals above certain BMI thresholds and is limited to specific age groups, which may restrict the external validity of our findings in severely obese populations.Added to the cardiovascular outcomes were self-reported, and may be influenced by recall bias. As well, renal risk classification was based on single measurements of eGFR and albuminuria, which may not adequately reflect transient changes. Furthermore, while our regression models were adjusted for key demographic and cardiometabolic variables, they did not account for other potential confounders available in NHANES, such as physical activity, alcohol consumption, socioeconomic status, or specific medication usage. This decision was made to maintain model parsimony and mitigate the risk of overfitting. We acknowledge that these unadjusted factors could potentially influence the observed associations, and future research incorporating a more comprehensive set of covariates is warranted to further delineate the independent contribution of MAFLD to CKM severity.

In conclusion, this analysis shows that MAFLD is strongly linked to CKM syndrome and its advancement, highlighting the observed strong association of the liver with the development of multi-organ metabolic disease and pointing out the shortcomings of a CKM framework that excludes liver-related metrics. As for improvement of risk assessment, it would be beneficial to incorporate liver health metrics, whether through elastography or validated non-invasive fibrosis scores, into CKM staging. This approach could lead to earlier identification of high-risk populations and facilitate integrated prevention strategies that focus on the liver-heart-kidney axis. As the global incidence of MAFLD continues to increase, modifying the existing CKM paradigm to include a cardiovascular-renal-hepatic-metabolic model could be a significant advancement in the comprehensive, multidisciplinary management of metabolic diseases.

## Supplementary Information

Below is the link to the electronic supplementary material.

Supplementary file1 (DOCX 26 KB)

## Data Availability

The datasets analysed during the current study are not publicly available due to ethical considerations, but are available from the corresponding author on reasonable request.
