# Supplementary material for: MAFLD increases CKM syndrome severity: a NHANES-based cross-sectional study
Source: Clin Exp Med. 2025 Dec 26;26(1):79. doi: 10.1007/s10238-025-01997-1 (PMC12769494; doi:10.1007/s10238-025-01997-1)
Supplement: Supplementary file 1 — Supplementary file1 (DOCX 26 KB) [file 10238_2025_1997_MOESM1_ESM.docx]

| CKM stages | Definition according to AHA 2023 | NHANES Variables |
| --- | --- | --- |
| Stage 0 (Healthy individuals) | Individuals without metabolic risk factors: normal BMI/WC, BP, glucose, and lipid; no CKD OR CVD | - BP (BPXDI1, BPXSY1)   - FBS (LBXGLU)   - Lipid (LBXTR, LBDHDD)  - BMI (BMXBMI)  - HTN (BPQ050A)  - DM (DIQ010)  Use fasting subsamples weight (WTSAFPRP) |
| Stage 1 | Individuals with visceral adiposity (high BMI/WC and insulin resistance (prediabetes) without other risk factors | -BMI (BMIXBMI)  - Insulin (LBXIN)  - Glucose (LBXGLU) |
| Stage 2 | Individuals with DM, HTN, Dyslipidemia or moderate-high-risk chronic CKD | -HTN (BPQ020, BPQ030)  -DM (DIQ010) or LBXGLU >126 mg/dl  -Lipid (LBXTR, LBXHDD)  -CKD (eGFR: LBXSCR, ACR: URXUMA, URXCRS) |
| Stage 3 | Individuals with metabolic syndrome and Subclinical CVD in individuals (> 20% ASCVD score or very high-risk CKD | -ASCVD: equation of Age, sex, race, SBP, Cholesterol, HDL, LDL, history of DM, smoking, hypertension drug (RIAGENDER, RIDAGEYR, LBXTC, LBXHDD, LBXLDL, BPXSY1, BPXDI1, BPQ50, BPQ020, SMQ020) |
| Stage 4 | Individuals with metabolic syndrome and clinical CVD (e.g., coronary heart disease, heart failure, stroke) | -Angina (MCQ160d), coronary heart disease (MCQ160c), heart failure (MCQ160e), and stroke (MCQ160f) |

CKM Syndrome framework-NHANES mapping

Abbreviations: CKM: cardiovascular-kidney-metabolic; BMI: body mass index; WC: waist circumference; BP: blood pressure; FBS: fasting blood sugar; DM: diabetes mellitus; CVD: cardiovascular disease; CKD: chronic kidney disease; ASCVD: Atherosclerosis cardiovascular disease.
